# Frequency and Seasonal Variations of Viruses Causing Respiratory Tract Infections in Children Pre- and Post-COVID-19 Pandemic in Riyadh (2017–2022)

**DOI:** 10.7759/cureus.33467

**Published:** 2023-01-06

**Authors:** Hanan Alaib, Najla Algariri, Hiba Ahmed, Amira Bebars, Fayza Alamri, Riad Durmush, Muhammad Ayaz, Walaeldin Hamadelnil, Badriah Alboriaki, Bader Altamimi, Mona Alalyani, Doaa SS Aljasser, Mohammed Aboud

**Affiliations:** 1 Pediatrics, King Abdulla Bin Abdulaziz University Hospital, Riyadh, SAU; 2 Pediatrics, University Hospital of North Tees, Stockton-On-Tees, GBR; 3 Pediatric Intensive Care Unit, King Abdulla Bin Abdulaziz University Hospital, Riyadh, SAU; 4 Pediatrics, Princess Nourah Bint Abdulrahman University, Riyadh, SAU; 5 Pediatric Complex Care, King Abdulla Bin Abdulaziz University Hospital, Riyadh, SAU; 6 Health Sciences Research Center, Princess Nourah Bint Abdulrahman University, Riyadh, SAU; 7 Pediatric Infectious Diseases, King Abdulla Bin Abdulaziz University Hospital, Riyadh, SAU

**Keywords:** respiratory tract infections, influenza virus type a and b, human rhino enterovirus (hre), respiratory syncytial virus (rsv), covid-19

## Abstract

Introduction

The COVID-19 pandemic has had a major impact on healthcare systems throughout the world. As the clinical and epidemiological features of COVID-19 share many similarities with other respiratory viruses in children, ensuring optimal management of different viral respiratory diseases is critical. The precautions taken to prevent COVID-19 have seemingly had an indirect effect on the seasonal variations of viral diseases and the frequency of relevant viruses. The seasonal irregularity of and uncertainty surrounding these infection peaks may affect the clinical prediction and management resources. Therefore, the aim of this study is to evaluate the impact of the COVID-19 pandemic on the frequency and seasonal variation of common respiratory viruses in children pre- and post-pandemic.

Methodology

This study utilizes a descriptive cross-sectional retrospective approach. A total of 726 samples collected from children below 14 years of age and admitted to King Abdulla bin Abdulaziz University Hospital between March 2017 and February 2022 were included in the present study to evaluate the impact of the COVID-19 pandemic on the frequency and seasonal variation of common respiratory viruses in children pre- and post-pandemic. The samples taken before March 15, 2020, were considered pre-COVID-19, and those taken from March 15, 2020, onward were considered post-COVID-19. The seasons were divided based on the months of the year as per the Saudi climate website (winter: December-February, spring: March-April, summer: May-August, and autumn: September-November).

Results

All nasopharyngeal swabs (NPS) for viral Polymerase chain reaction (PCR) multiplex that were done for all admitted children of age up to 14 years were included, and the total samples amounted to 726, There were 686 (94.4%) positive samples for viruses and 40 (5.5%) negative samples. The number of positive samples pre-COVID-19 pandemic was 494 (72%), and the number of positive samples post-COVID-19 pandemic was 192 (28%). The frequency of different viruses has decreased post-COVID-19 and seasonality has changed; Although Adenovirus, and influenza viruses have no big changes, but Human Rhino/enterovirus (HRE) has increased frequency post-COVID-19 (49%), while post-COVID-19 it was (29.1%). The seasonal peak for Respiratory Syncytial Virus (RSV) pre-COVID-19 showed mainly in winter (49%), while post-COVID-19 it showed no peak.

Conclusion

The frequency of most types of viruses is noted to be lesser in the post-COVID-19 period, most likely due to precautions followed during the pandemic. This is not the case for HRE which showed increasing frequency in post-COVID-19; However, there are clinically and statistically significant differences among seasonal peaks in Respiratory RSV, HRE, and Parainfluenza viruses (PIV) pre- and post-COVID-19 pandemic. RSV showed no peak in different seasons post-COVID-19, although its peak pre-COVID-19 was in winter and autumn; Additionally typical trend of HRE peak changed to be in Autumn and spring post-COVID-19 instead of winter pre-COVID-19.

## Introduction

The COVID-19 pandemic has had a major impact on healthcare systems throughout the world. As the clinical and epidemiological features of COVID-19 share many similarities with other respiratory viruses in children, ensuring optimal management of different viral respiratory diseases is crucial. The precautions followed to prevent COVID-19 (including travel restrictions and closure of workplaces and schools, as well as social distancing, mask usage, and hand hygiene) have an indirect effect on the seasonal variation of viral diseases and the frequency of relevant viruses. 

Some studies showed that both in Europe and Australia, only a few RSV cases have been detected even after the removal of the most restrictive measures, i.e., when only hand washing, social distancing, and mandatory face mask usage were maintained [1,2].

Despite the above-mentioned precautions, rhinovirus cases were noted to increase in frequency during the COVID-19 pandemic while the frequency of infections caused by enveloped viruses (influenza A; human metapneumovirus; human parainfluenza virus types 1, 2, 3, and 4; and human respiratory syncytial virus) decreased.

The seasonal irregularity of and uncertainty regarding these infection peaks may affect the clinical prediction and management resources. This research could help in developing a prospective therapeutic approach for viral respiratory tract infections in children.

The objective of this research was to describe the seasonal variation of common respiratory viruses in children pre- and post-COVID-19-pandemic and identify the frequency of each virus causing respiratory tract infections in each season and determine their peak pre- and post-COVID-19-pandemic.

## Materials and methods

Study design

The present study was designed as a descriptive cross-sectional retrospective study. A total of 726 samples taken from children below 14 years of age and admitted to King Abdulla bin Abdulaziz University Hospital between March 2017 and February 2022 were included in our study to evaluate the impact of the COVID-19 pandemic on the frequency and seasonal variation of common respiratory viruses in children pre- and post-pandemic. The samples taken before March 15, 2020, were considered pre-COVID-19, and those taken from March 15, 2020, onward were considered post-COVID-19. The seasons were divided based on the months of the year as per the Saudi climate website (winter: December-February, spring: March-April, summer: May-August, and Autumn: September-November).

Data collection

Data were collected from patient medical records through the electronic system. All nasopharyngeal swabs (NPS) were collected upon admission. The swabs were tested via respiratory multiplex PCR for 12 viruses and four bacteria (respiratory syncytial virus [RSV], adenovirus [ADV], coronavirus 229E, coronavirus HKU1, coronavirus NL63, coronavirus OC43, MERSCOV, SARS-CoV-2 (COVID-19), human metapneumovirus (HMPV), human rhino/enterovirus (HRE), influenza A (AH1, AH3, AH2009), influenza B, parainfleunza [PIV] [1-4], Bordetella pertussis, Bordetella parapertussis, Chlamydia pneumoniae, and Mycoplasma pneumonia); then, these data that include the medical file number, date of admission, date of discharge, age upon admission, diagnosis upon admission, diagnosis upon discharge, length of stay, Pediatric Intensive Care Unit (PICU) admission, PICU length of stay, type of respiratory support and oxygen concentration, need for chest tube, nasopharyngeal swabs results, chest x-ray findings, investigations results including for CBC, CRP, procalcitonin, and cultures, need for steroid, need for antibiotics, and readmission within seven days were entered in REDCap system, cleaned up, and then exported to an Excel sheet.

Statistical analysis

Data were analyzed using JMP version 14. Descriptive data were presented as mean ± standard deviation for the continuous variables. Categorical variables were reported as frequency (percentage). Chi-square analyses were used to test the association between the COVID-19 pandemic and the seasonal variations of different viruses causing respiratory diseases in children.

## Results

In total, there were 726 samples, divided per season (autumn, spring, summer, winter), as shown in Table 1. There were 686 (94.4%) positive samples for viruses and 40 (5.5%) negative samples. The number of positive samples pre-COVID-19 pandemic was 494 (72%), and the number of positive samples post-COVID-19 pandemic was 192 (28%).

**Table 1:** Demographic data

Characteristics	Pre-COVID-19	%	Post-COVID-19	%	P-value
AGE					
≤ 28 days	29	6	14	7	0.924
29-90 days	84	16	30	15	
3month-12month	145	28	60	29	
>1yr - 2yrs	105	20	37	18	
>2yrs-6yrs	123	24	52	25	
>6 yrs	35	7	12	6	
Total	521	100	205	100	
GENDER					
Male	261	50	127	62	0.0038
Female	260	50	78	38	
Total	521	74.5	205	100	
SEASON					
Autumn	133	25.5	49	23.9	0.0001
Spring	67	12.9	41	20	
Summer	115	22	67	32.7	
Winter	206	39.5	48	23.4	
Total	521	100	205	100	

Regarding the frequency of each virus pre- and post-COVID-19 pandemic as shown in Table 2 we found the typical picture pre-COVID-19 was changed at post -covid-19 period, Respiratory Syncytial Virus (RSV) frequency much decreased post-COVID-19, it was seen in 189 samples (38.25%) pre-COVID-19 and in 27 samples (14%) post-COVID-19. Adenovirus (ADV) showed no change in both periods, it represented 9.5% and 9.4% pre- and post-COVID-19, respectively, Human metapneumovirus (HMPV) constituted 3.84% of the total positive cases pre-COVID-19 and came up to 5.2% in post-COVID-19 positive samples. Meanwhile, HRE was found in 144 (29.1%) samples pre-COVID-19 and in 94 (49%) samples post-COVID-19. Influenza A (IA) represented 9.1% pre-COVID-19 and 3.1% post-COVID-19 and influenza B (IB) constituted 2.83% and 1.6% pre-COVID-19 and post-COVID-19, respectively, while parainfluenza virus (PIV) was detected in 36 positive samples (7.29%) pre-COVID-19 and in 11 (5.7%) samples post-COVID-19. So, with this data, we noticed in particular that enveloped viruses like RSV and influenza viruses much decreased in overall their frequency, while HRE viruses were coming up post-COVID-19, while some viruses like ADV have not much changed across whole years (Table 2).

**Table 2:** Total number of common respiratory viruses pre- and post-COVID-19 pandemic

Virus	Pre-COVID-19	%	Post-COVID-19	%
Respiratory Syncytial Virus (RSV)	189	38.25	27	14
Adenovirus (ADV)	47	9.5	18	9.4
Human metapneumovirus (HMPV)	19	3.84	10	5.2
Human Rhino/Enterovirus (HRE)	144	29.1	94	49
Influenza B (IB)	14	2.83	3	1.6
Influenza A (IA)	45	9.1	6	3.1
Parainfluenza Virus (PIV)	36	7.29	11	5.7
Severe Acute Respiratory Syndrome Coronavirus 2 (SARS-COV2)	0	0	23	12
TOTAL	494	100	192	100

**Figure 1:**
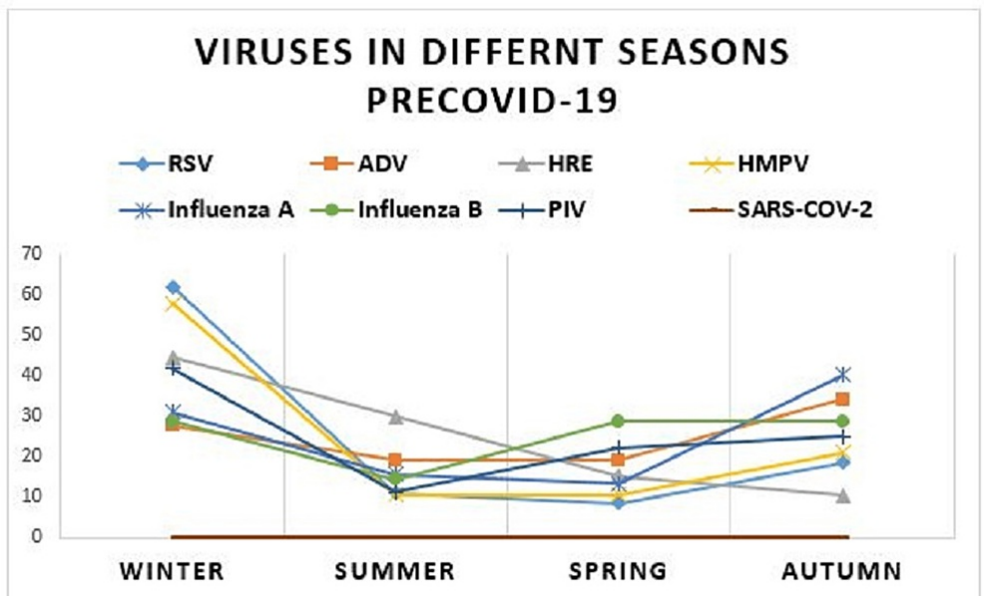
Seasonal peaks for viruses pre-COVID-19

**Figure 2:**
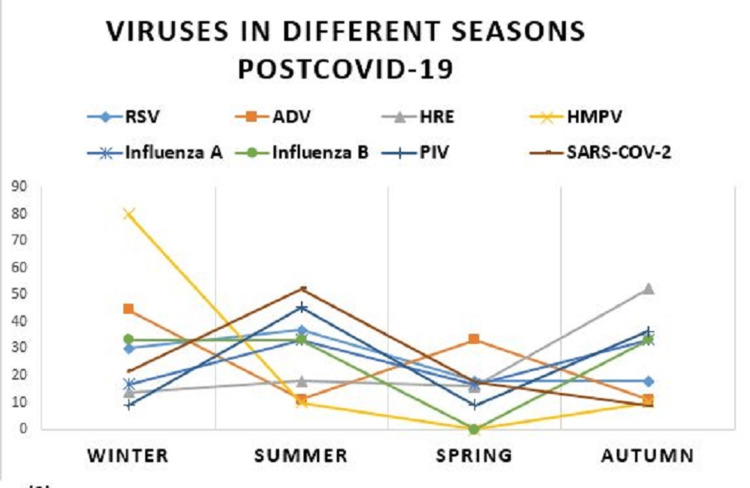
Seasonal peaks for viruses post-COVID-19

The typical trend of viruses’ frequency at different seasons, was completely subverted in the post-COVID-19 period. RSV represented 34.7% of the viruses in autumn pre-COVID-19 and 7.6% in autumn post-COVID-19. In winter, it constituted 49% pre-COVID-19 and 15.9% in winter post-COVID-19. In summer, it represented 23.8% and 20% pre-COVID-19 and post-COVID-19, respectively, and in spring, it amounted to 23.9% and 15.6% pre-COVID-19 and post-COVID-19, respectively (P-value = 0.0008). So, we have not witnessed typical winter season of RSV post-COVID-19 (Table 3, Figures 1, 2).

**Table 3:** Seasonal variations in common viruses causing respiratory infection in children

viruses	Autumn	Spring	Summer	Winter	P-value
RSV					
Pre-COVID-19	35(34.7%)	16(23.9%)	21(23.8%)	117(49%)	0.0008
Post-COVID-19	5(7.6%)	5(15.6%)	10(20%)	7(15.9%)	
ADV					
Pre-COVID-19	16(15.8%)	9(13.4%)	9(10.2%)	13(5.4%)	0.132
Post-COVID-19	2(3%)	6(18.75%)	2(4%)	8(18.1%)	
HRE					
Pre-COVID-19	15(14.9%)	22(32.8%)	43(48.8%)	64(20.9%)	0.049
Post-COVID-19	49(74%)	15(46.9%)	17(3.4%)	13(29.5%)	
HMPV					
Pre-COVID-19	4(3.96%)	2(2.9%)	2(2.2%)	11(4.6%)	0.444
Post-COVID-19	1(1.5%)	0 (0%)	1(2%)	8(18.1%)	
IB					
Pre-COVID-19	4(3.96%)	4(5.9%)	2(2.2%)	4(1.68%)	0.7048
Post-COVID-19	1 (1.5%)	0(0%)	1(2%)	1(2.27%)	
IA					
Pre-COVID-19	18(17.8%)	6(8.9%)	7(7.9%)	14(5.9%)	0.6993
Post-COVID-19	2 (3%)	1(3.1%)	2(2%)	1(2.27%)	
PIV					
Pre-COVID-19	9 (8.9%)	8(11.9%)	4(4.5%)	15(17%)	0.0297
Post-COVID-19	4 (6%)	1(3.1%)	5(10%)	1(2.27%)	
SARS-COV 2					
Pre-COVID-19	0	0	0	0	
Post-COVID-19	2 (3%)	4 (12.5%)	12 (24%)	5 (11.36%)	
Total					
Pre-COVID-19	101	67	88	238	
Post-COVID-19	67	32	50	44	

ADV represented 15.8% and 5% in autumn pre-COVID-19 and post-COVID-19, respectively, and it constituted 5.4 % and 18.1% in winter pre- and post-COVID-19, respectively. In summer, it amounted to 10.2% and 4% pre- and post-COVID-19, respectively, and in spring, ADV representation pre-COVID-19 was 13.4% and 18.75% post-COVID-19 (P-value = 0.132).

HRE has major changed in its seasonality, it came up in autumn and spring post-COVID-19; in pre-COVID-19 autumn constituted 14.9% and 75% in post-COVID-19 autumn, and in winter, pre-COVID-19, it represented 26.9%, and in winter, post-COVID-19, it amounted to 29.5%. Further, in summer, it represented 48.8% pre-COVID-19 and 3.4% in post -COVID-19 summer. In spring, HRE was at 32.8% pre-COVID-19 and at 46.9% in spring post-COVID-19 (P-value = 0.0490).

HMPV, IB, and IA viruses showed no great changes in their pattern. This could be related to small samples which cannot reflect the real situation of these viruses. We noticed HMPV in pre-COVID-19 autumn it represented 3.96%, while in post-COVID-19 autumn, it constituted 1.5%. In the pre-COVID-19 winter, it was positive in 4.6% of the positive samples, while in the winter post-COVID-19, it was positive in 18.1%. Further, in pre-COVID-19 summer, it represented 2.2%, and in post-COVID-19 summer, it amounted to 2%. In spring pre-COVID-19, it was at 2.9%, while in post-COVID-19 spring, it was at 0% (P-value = 0.444).

IB in autumn pre-COVID-19 represented 3.96%, while it was positive in 1.5% of the total positive samples in autumn post-COVID-19. In pre-COVID-19 winter, it constituted 1.68% while in winter post-COVID-19, it came to 2.27%. In pre-COVID-19 summer, it was at 2.2%, while in post-COVID-19 summer, it was 2%. In spring, it represented 5.9% and 0% in pre-COVID-19 and post-COVID-19, respectively (P-value = 0.7048).

IA in autumn pre-COVID-19 represented 17.8%, while it was positive in 3% of the total positive samples in post-COVID-19 autumn. In pre-COVID-19 winter, it was at 5.9% while in winter post-COVID-19, it was at 2.27%. In pre-COVID-19 summer, it represented 7.9%, and in post-COVID-19 summer, it constituted 2%. In spring, it came to 8.9% and 3.1% in pre-COVID-19 and post-COVID-19, respectively (P-value = 0.6993).

Although a small sample size for PIV, it showed an overall decreased and no winter peak in post-COVID-19. It was in pre-COVID-19 autumn represented 8.9% and 6% in post-COVID-19 autumn. In pre-COVID-19 winter, it was at 17%, and in winter post-COVID-19, it was at 2.27%. In summer, pre-COVID-19, it represented 4.5%, and in post-COVID-19 summer, it constituted 10%. In spring pre-COVID-19, PIV was at 11.9%, and in spring post-COVID-19, it came down to 3.1%, (P-value = 0.297). SARS-CoV-2 in pre-COVID-19 across all seasons represented 0% and constituted 3% in post-COVID autumn, 11.36% in winter, 24% in summer, and 12.5% in spring.

## Discussion

The present paper shows that most of the viruses causing respiratory illnesses in children decrease post-COVID-19 compared with the pre-COVID-19 period. However, the HRE virus showed the opposite, and a mild increase in HMPV is seen. Meanwhile, ADV showed almost no change, which could be related to the non-pharmacological intervention (NPI) (use of masks, social distancing, closure of workplaces and schools, hand hygiene, etc.).

Previous studies have reported that viral interference among influenza viruses, rhinovirus, and other respiratory viruses can affect viral infections at the host and population levels. In this study, rhinovirus infection considerably increased in children despite the recommended precautions. Since rhinovirus is a non-enveloped virus, it is relatively resistant to ethanol-containing disinfectants [3], and it can survive on environmental surfaces for a prolonged period of time [4]. And this might be due to the decreasing percentage of influenza viruses; Wu et al. stated Rhinovirus infection induces an antiviral interferon response that protects against influenza A virus infection in human airway epithelial cells [5]. Furthermore, Leung et al. reported that surgical face masks could prevent the transmission of seasonal human coronaviruses and influenza viruses, but not that of rhinoviruses, from symptomatic individuals [6].

However, Takashita et al. reported that after May 2020, they did not detect enveloped viruses (i.e., influenza virus; human metapneumovirus; human parainfluenza virus types 1, 2, 3, and 4; and human respiratory syncytial virus). However, they did detect non‐enveloped viruses, including rhinovirus; Coxsackievirus A and B; and human adenovirus. This disparity in detection between enveloped and non‐enveloped viruses might be related to their stability [7]. Williams et al. stated in their study that in the absence of a usual autumn/winter seasonal pattern for RSV, countries in both hemispheres have experienced a range of subsequent epidemic trajectories [8].

This paper shows COVID-19’s effect on the peak incidence of RSV, influenza A, HRE, and PIV. RSV showed no peak post-COVID-19, while its peak pre-COVID-19 was in winter. HRE’s peak changed to autumn instead of winter during the pre-COVID-19 period. IA was noted in winter; however, post-COVID-19, it had two peaks in summer and autumn, while PIV’s peak was in winter and in summer post-COVID-19. The United States Centers for Disease Control and Prevention (CDC) has also reported an increase in RSV activity in May to June 2021 [7], which is unusual for this period.

In contrast, in Australia, the percent of influenza tests that were positive continued to be very low (<0.01%), indicating limited influenza transmission in the community. Since early March 2020, this percentage has remained far lower than the usual range for the time of year [9]. Similarly, the US reported in May 2020 that the influenza hospital rates were 90% lower than during the low severity 2011/12 season with persistently low rates across the autumn/winter of 2020/21 [10].

Although the World Health Organization (WHO) and European Centre for Disease Prevention and Control (ECDC) have reported that influenza activity during the 2020/2021 influenza season did not increase above baseline with no indication of an autumn/winter spike, despite widespread and regular testing for influenza viruses, this has continued into the spring and summer of 2021, where influenza activity remained at inter-seasonal levels [10].

This paper has limitations include samples were from one center; however, we tried to expand our sample size by include all cases with positive NPA PCR Pre and Post COVID-19. Our study has some limitation which is small sample sizes at post-COVID-19 period and it was done in one center, we tried to partially compensate this issue, by including the whole years since our center start to work, and by doing the analysis for each period separately, then comparing virus' percentage not numbers.

## Conclusions

The frequency of most types of viruses is noted to be lesser in the post-COVID-19 period, most likely due to precautions followed during the pandemic. This is not the case for HRE which showed increasing frequency post-COVID-19. However, there is clinically and statistically significant differences among seasonal peaks in RSV, HRE, and PIV viruses pre- and post-COVID-19 pandemic. RSV showed no peak in different seasons post-COVID-19, although its peak pre-COVID-19 was in winter and autumn. Additionally, the typical trend of HRE peak changed to be in autumn and spring post-COVID-19 instead of winter pre-COVID-19.
